# Cajeput Oil in Cholera

**Published:** 1831

**Authors:** 


### CAJEPUT OIL IN CHOLERA.

This stimulating oil is beginning to attract attention, and to throw in its claim for notoriety. How far and how long it may be able to supersede or eclipse the long-established reputation of opium, brandy, and calomel in the painful spasms, and disordered secretions of our autumnal visitations of cholera, we will not pretend to say.

Sir Matthew Tierney and Mr. Bushell, have published some cases where the cajeput oil was considered to be very advantageous; but whoever has seen much of cholera must be well aware of the sudden relief which is often afforded by hot and aromatic substances of every kind—by spices, and by brandy.

I. The following is the case published by Sir M. Tierney, in our contemporary, the Medical Gazette.

"A lady, æt. 28, felt indisposed at half-past nine, P. M. on Wednesday, the 10th instant: she had been in good health throughout the day, and dined, as usual, at eight o'clock. At a little before ten vomited the contents of the stomach, reported to be merely the food taken at dinner: the bowels were moved shortly after. At half-past ten the vomiting and purging again took place, and she felt "very uncomfortable." Continued occasionally purged and sick at the stomach till a little before one, when she fainted, and remained insensible for about ten minutes: on recovering, she was seized with violent spasms in the lower extremities, more particularly in the feet, the toes being remarkably affected. The nausea and vomiting again distressing.

On my visiting this lady (for the first time since the invasion of the symptoms,) at a quarter before two, A. M. on Thursday, the 11th, I found her in a profuse perspiration, with a death-like coldness of the extremities; the pulse at the wrist scarcely perceptible; insatiable thirst; countenance expressive of great anxiety, with a remarkable shrinking of the features; and extreme restlessness: the mind perfectly clear. She said, 'I believe that I have got the cholera: I took twenty-five drops of the cajeput oil about half an hour ago, and, in a few minutes after, fifty drops more: it has done me good; pray let me have another dose.' I assented, and, fearing that what she had already taken might not have been genuine, I sent for some which I had received from a friend lately arrived from India: in the meantime, she took three tea-spoonfuls of brandy, in a little water, which was repeated in five minutes. The body and limbs having been well rubbed with hot, dry flannels, were wrapped up in the same: this was attended with considerable difficulty, from the great restlessness and jactitation.

At two o'clock, A. M. I gave forty drops of the oil, in half a wine-glass of warm water: this at once quieted the stomach, and in half an hour the spasms were somewhat relieved: the pulse became more perceptible, and she said that she felt better; but the thirst continued unabated, and she called for iced water, of which she was permitted to take half a wine-glass repeatedly, with the addition of a small quantity of brandy, and a little sugar.

At half-past three the extremities became quite warm, indeed they were rather above the natural temperature; but the restlessness was at this time excessive, and a stool was passed, consisting of about six ounces of fluid resembling thick rice-water. Plain water, soda-water, lemon-peel water, iced, with a little brandy, occasionally given. The feeling of weakness excessive: there was a disposition to sleep, but this was interrupted by extreme thirst: the stomach and bowels now quiet. At five o'clock she anxiously requested a saline effervescing draught, which was given, but immediately rejected by the stomach: the pulse became more languid; another dejection, similar to the last; hiccup; the spasms increased in violence, and she complained of excruciating pains across the loins. Took ten drops of laudanum, with ten grains of Epsom salts, in a little peppermint water, but this was soon rejected, and not followed by any alleviation of the symptoms.

At six o'clock, A.M. I had the pleasure of having Dr. Holland associated with me. A blister was directed to be applied to the epigastric region; a draught ordered, containing a small quantity of Epsom salts in a little peppermint-water, which was immediately rejected by the stomach; the small quantity of brandy in iced water, soda water, &c. directed to be given occasionally.

The thirst continuing unabated, the patient was permitted to have small bits of ice in her mouth, which gave great comfort. During all this time *no urine was passed;* throughout there was no pain in the stomach or bowels.

The extremities becoming again cold, and the pulse giving way, it was agreed, at eight o'clock, to give forty drops of the cajeput oil. Great relief followed the exhibition of this dose; within an hour, however, the violent spasms in the muscles of the legs and feet returned, but they lasted only for a short time; she then became composed, and soon after had a short refreshing sleep.

At twelve o'clock great improvement; and at two, P.M. (the stomach and bowels having remained quiet) she took two grains of calomel, with three of the compound extract of colocynth. At three o'clock the patient took a small breakfast-cup of mulagatawney soup; and had afterwards refreshing sleep for an hour and a half; at five took a wine-glass of sherry, and slept again for an hour; at seven took another cup of the soup, and afterwards slept till half-past eight, when a dark, scanty stool passed; and for the first time since the attack, some urine; at half-past nine another scanty motion, and soon after a greater quantity of urine, of a pale colour; at ten o'clock, P.M. she took four grains of the extract of colocynth, and she was removed to a sofa while her bed was being made; at half-past ten, after taking a little more soup, she fell asleep.

Friday, 12th, nine, A.M.—I found the patient asleep; her maid reported that she had passed a good night, and slept comfortably; urine passed freely; at noon met Dr. Holland; we found our patient free from complaint, having taken some of the soup for her breakfast. It is to be noted, that throughout this attack the tongue was clean and moist, although the thirst at times was intolerable. In the course of this day she was moved from her bed to a sofa; at nine in the evening, the bowels not having been moved, eight grains of the extract of colocynth were taken.

Saturday, 13th, morning. — Found that the patient had been disturbed by the pills during the night, and had had loose bilious motions.

Sent for at five, P.M. in consequence of her suffering occasional pain in the stomach and bowels, with vomiting. A draught, consisting of camphor, julep, and opiate confection, was ordered, but this was immediately rejected by the stomach. The pain and sickness continuing, thirty drops of the cajeput oil were administered, and soon after the pain and sickness subsided. The cajeput oil was always very grateful to the stomach. The lady has continued well. No other case of cholera has occurred in the family."

Considering the protracted state of indisposition in this case, we are very far from believing the cajeput oil was a better medicine than many others which we could mention. However, we do not wish to detract from the utility of the medicine, if it be possessed of any good qualities. It is a very old acquaintance of ours in the East, though seldom employed except externally.

II. Mr. Bushell, an intelligent practitioner, has published, through the same channel, two cases treated by the same remedy. They are as follow:—

"August 20th, 1831.—Hannah ——, aged 50, housemaid, is subject to dyspepsia, but has been well until between seven and eight this evening, when she was seized suddenly with considerable abdominal pain, diarrhœa, and vomiting. I was called to her about ten, and found the vomiting severe and incessant, of a clear transparent mucus. Bowels purged five times; motions dark, fluid, and offensive; pain of abdomen intense, with much tenderness, and the muscles apparently in a state of cramp, or spasm; extreme anxiety; cold clammy perspiration; pulse very weak, about eighty. I prescribed as follows:

℞. Olei Cajeputi guttas, xl.

Magnesiæ Subcarbonatis, ℈j.

Aquæ Pur. ℥iss. M. f. haustus stat. sum.

11 o'clock.—The relief from the medicine has been decided, to use my patient's words, 'to perfection.' The vomiting and pain in the bowels have subsided, and she complains only of being very weak and cold.

℞. Ol. Cajeputi guttas, xl.

Magnesiæ Subcarb. ʒss.

Aquæ Puræ, ℥vj. M. f. mistura sumat 4tam partem 2dis horis.

August 21st.—This morning I find my patient free from complaint; she will continue the medicine at longer intervals. She has had an alvine evacuation during the day, of a natural colour.

Sunday, Aug. 21st.—About 10 o'clock this evening I was sent for to Mrs. H. aged 35, about five months advanced in pregnancy. I found her complaining of excruciating abdominal pain, with vomiting of clear transparent mucus, and frequent purging; her pulse very weak, 75. She had taken about two table-spoonsful of brandy about half an hour previous to my visit, but this was instantly rejected by the stomach. The tenderness on pressure over the abdomen was so exquisite, and accompanied by pregnancy, that, had it not been for the experience of last night, I certainly would have bled largely. I determined, however, on giving the ol. cajeputi, in the dose of forty-five drops, combined with twenty grains of magnesia, in water. I saw my patient again at eleven. The effect of the medicine has been very beneficial: the pain had much subsided, and the stomach and bowels were quiet. I have directed fifteen drops to be taken every two hours during the night.

August 22d.—This morning I find her full of spirits, having taken three doses of the medicine.

The cajeput oil may be most gratefully administered combined with magnesia, which has the property of diffusing or causing essential oils to dissolve in water, and which, in the opinion of Annesley, has a certain degree of power itself over the stomach, in cases of cholera. I am disposed to think that the cajeput has some influence over the symptoms of the present epidemic; but that it has more than many other volatile oils (more especially peppermint), remains, I think, with Dr. Macleod, in your number of the 13th of this month, to be proved. I remember reading in Johnson's Medico-Chirurgical Review, some years ago, accounts of its very great efficacy in the treatment of the Indian cholera; but, from want of an Index to that truly valuable periodical, I cannot place my hand on the number, but so far as I can charge my memory the doses were large — about one hundred drops. So much has been said, and from such high authority (Sir M. Tierney), on the powers of the cajeput over cholera, that the drug monopolizers are at work, and its price has fully doubled within the last fornight.

THOMAS BUSHELL."

